# Cellular senescence and aging: the role of B-MYB

**DOI:** 10.1111/acel.12242

**Published:** 2014-07-01

**Authors:** Sophia N Mowla, Eric W-F Lam, Parmjit S Jat

**Affiliations:** 1 Department of Neurodegenerative Disease and MRC Prion Unit, UCL Institute of NeurologyQueen Square, London, WC1N 3BG, UK; 2 Division of Cancer, Department of Surgery and Cancer, Imperial Centre for Translational and Experimental Medicine, Imperial College London, Hammersmith HospitalDu Cane Road, London, W12 0NN, UK

**Keywords:** aging, B-MYB, cellular senescence, growth arrest, MuvB

## Abstract

Cellular senescence is a stable cell cycle arrest, caused by insults, such as: telomere erosion, oncogene activation, irradiation, DNA damage, oxidative stress, and viral infection. Extrinsic stimuli such as cell culture stress can also trigger this growth arrest. Senescence is thought to have evolved as an example of antagonistic pleiotropy, as it acts as a tumor suppressor mechanism during the reproductive age, but can promote organismal aging by disrupting tissue renewal, repair, and regeneration later in life. The mechanisms underlying the senescence growth arrest are broadly considered to involve p16^INK4A^-pRB and p53-p21^CIP1/WAF1/SDI1^ tumor suppressor pathways; but it is not known what makes the senescence arrest stable and what the critical downstream targets are, as they are likely to be key to the establishment and maintenance of the senescent state. MYB-related protein B (B-MYB/MYBL2), a member of the myeloblastosis family of transcription factors, has recently emerged as a potential candidate for regulating entry into senescence. Here, we review the evidence which indicates that loss of B-MYB expression has an important role in causing senescence growth arrest. We discuss how B-MYB acts, as the gatekeeper, to coordinate transit through the cell cycle, in conjunction with the multivulval class B (MuvB) complex and FOXM1 transcription factors. We also evaluate the evidence connecting B-MYB to the mTOR nutrient signaling pathway and suggest that inhibition of this pathway leading to an extension of healthspan may involve activation of B-MYB.

## Introduction

Cellular senescence, originally described as a form of cell aging, is a stable cell cycle arrest caused by insults including telomere erosion, oncogene activation, irradiation, DNA damage, oxidative stress, viral infection, and toxins (Ben Porath & Weinberg, 2004; Collado *et al*., 2007; Vaughan & Jat, 2011; López-Otín *et al*., 2013). Extrinsic stimuli such as cell culture stress can also trigger this programmed growth arrest. These stressors induce a complex stress response that results in the activation of two well-known tumor suppressors, p53 and pRB (retinoblastoma susceptibility protein; Ryan *et al*., 2001). Upon activation, p53 upregulates the cyclin-dependent kinase inhibitor, p21^CIP1/WAF1/SDI1^, that inhibits the Cyclin A-, Cyclin E-, and Cyclin D-dependent kinase complexes (Lowe & Sherr, 2003). CyclinD-CDK4/6, CyclinE-CDK2, and CyclinA-CDK2 normally phosphorylate and inactivate pRB, and this is prevented by p21^CIP1/WAF1/SDI1^ (Lowe & Sherr, 2003). Dephosphorylated pRB represses the G1/S transition by sequestering E2F transcription factors, thereby inhibiting E2F-dependent gene expression (Ben-Porath & Weinberg, 2005; Di Micco *et al*., 2008). The p107 and p130 pRB-like pocket proteins associate with E2Fs and MuvB core proteins to form the DREAM [dimerization protein (DP), RB-like, E2F, and MuvB] complex that represses all cell cycle-dependent genes upon quiescence (Sadasivam *et al*., 2012; DeCaprio, 2013; Sadasivam & DeCaprio, 2013). The pRB pathway may also be activated independently of p53 via upregulation of p16^INK4A^, which inhibits CyclinD–CDK4/6 complexes from specifically phosphorylating and inactivating pRB (Rufini *et al*., 2013). The tumor suppressor, p53, is a master transcription factor that exerts control over global transcriptional networks and therefore also induces senescence by alternative pathways (Rufini *et al*., 2013). Persistent activation of the p53 and pRB pathways results in cellular senescence, a phenomenon commonly associated with biological aging.

## Senescence, cancer, and aging

Senescence is thought to have evolved as an example of antagonistic pleiotropy, as it provides beneficial traits during the reproductive age of an individual, but confers deleterious effects later on in life (Campisi, 2005, 2013). This is because, when organisms are young, senescence acts as a tumor suppressor mechanism through the removal of genetically unstable cells or cells that have acquired an oncogenic mutation, by inhibiting their proliferation (oncogene-induced senescence) (Campisi, 2005, 2013); replicative immortality gained through the bypass of cellular senescence is thus a key hallmark of cancer cells. However, later in life, senescence promotes organismal aging via the disruption of tissue renewal, repair, and regeneration and the accumulation of nondividing (senescent) cells (Kuilman *et al*., 2008).

The loss of proliferative potential with age should suppress cancer, but cancer incidence, like other degenerative diseases of aging, increases nearly exponentially with age. There is now increasing evidence that the increase in cancer incidence is due to senescent cells secreting factors that create a tissue microenvironment that promotes tumor formation. Senescent cells secrete a plethora of inflammatory cytokines, chemokines, and matrix metalloproteinases such as stromelysin (MMP3), as part of the senescence-associated secretory phenotype (SASP) proteins. The SASP proteins promote epithelial-to-mesenchymal transition as well as the degradation of basement membranes, thereby increasing cell migration, invasion, and metastasis. As a result, this activates immune cells causing the engulfment of surrounding tissue, resulting in tissue degradation (Tchkonia *et al*., 2013). Senescent cells also secrete factors such as WNT16B, interleukin-6 (IL-6), and tissue inhibitor of metalloproteinases-1 (TIMP-1) that can protect neighboring tumor cells from being killed (Gilbert & Hemann, 2010; Sun *et al*., 2012). SASP inflammatory proteins can also induce areas of local inflammation; cumulatively, this promotes an aging pathology and chronic age-related diseases through the disruption of tissue architecture and function (Longo & Finch, 2003; Kuilman *et al*., 2008). Senescence has been strongly associated with age-related diseases such as type II diabetes, cardiovascular disease, and neurodegenerative disease, three of the world’s largest killers (Golde & Miller, 2009; Khan *et al*., 2012; Monickaraj *et al*., 2013). A meta-analysis of genome-wide association studies and age-associated diseases has identified two highly significant peaks of disease association, mapping to the major histocompatibility locus and the INK4/ARF locus (Jeck *et al*., 2012). Senescence has hence been flagged as a novel target against aging and its related pathologies, as it may potentially be more beneficial to treat the underlying cause of these diseases (aging) rather than treating their symptoms individually. This has led to the idea that antisenescence therapy could lead to an extension of human healthspan. Although in its infant stage, the promotion of antisenescence mechanisms has already been shown to decrease the onset of age-related pathology *in vivo*, whilst the clearance of p16^INK4A^-positive senescent cells leads to an increase in healthspan (Longo & Finch, 2003; Baker *et al*., 2011; Huang *et al*., 2011). However, little is known about the critical molecular targets, downstream of p53 and pRB, that are specific to the senescent cell fate and what makes the senescence growth arrest stable. Moreover, much of the focus behind defining such targets has been around genes that are upregulated during senescence and are direct inducers, rather than genes that are inhibited during this process; such knowledge is imperative to the development of therapeutics and strategies that ameliorate the degenerative pathologies of aging.

## B-MYB

A recently emerging candidate that seems to play a role in attenuating senescence is B-MYB. B-MYB, a member of the myeloblastosis family of transcription factors, is present in all vertebrates and has vital antisenescence qualities due to its role in cell proliferation and growth (Martinez & DiMaio, 2011). Transcription of B-MYB occurs mainly at the onset of S-phase and is functionally activated by the CyclinA–CDK2 complex (Lefebvre *et al*., 2010; Down *et al*., 2012). The MYB family of ubiquitously expressed transcription factors (TFs) is comprised of three isoforms, A-MYB, B-MYB, and C-MYB. C-MYB was the first to be discovered when it was found to be the oncogene present in avian myeloblastosis virus, which causes acute myeloblastic leukemia and can transform hematopoietic cells in culture; A- and B-MYB were subsequently cloned via homology to C-MYB (Roussel *et al*., 1979; Lam *et al*., 1992). The literature surrounding MYB proteins is therefore skewed toward their oncogenic potential; very little attention has been focused on their antisenescence properties particularly those of B-MYB.

MYB isoforms have distinct biological roles, as demonstrated by their dissimilar patterns of gene expression (Rushton *et al*., 2003). One property, crucial to the exclusivity of B-MYB and its antisenescence role, is that unlike the other MYB isoforms, B-MYB is expressed in all replicating cells and is strongly associated with cell cycle progression (Ness, 2003). This is related to it being regulated transcriptionally by the pocket proteins and E2F transcription factors (Lam & Watson, 1993), as well as its ability to interact with the MuvB core complex (Quaas *et al*., 2012).

### The DREAM complex and cell cycle progression

**Figure 1:**
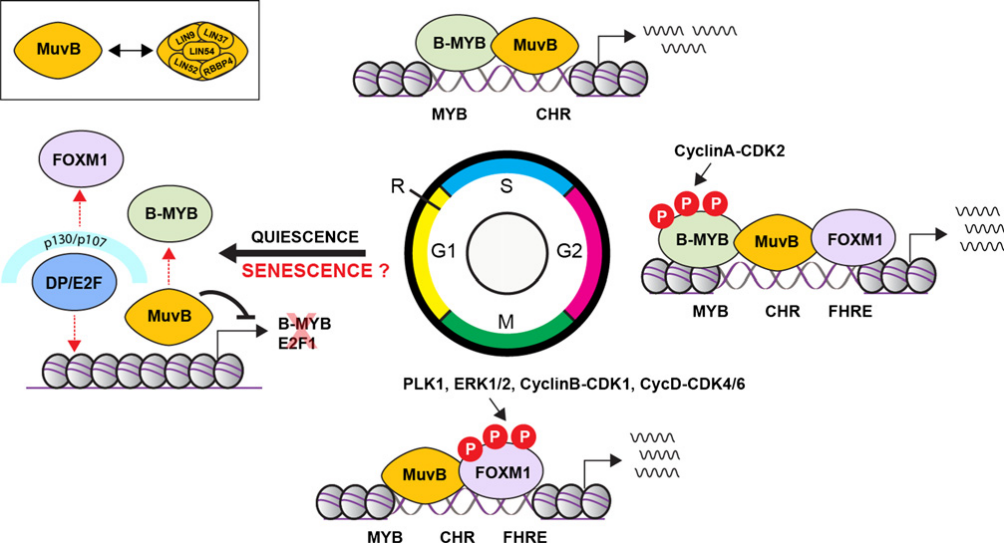
A model for how the MuvB core complex interacts with B-MYB, FOXM1, and p130–E2F4–DP1 to regulate cell cycle progression and entry into senescence. The MuvB core complex functions as a cofactor/adaptor for different transcription complexes to regulate gene expression. In quiescence, p130/p107 associates with E2F4 and DP to form the DREAM complex that represses all cell cycle-dependent gene expression (Sadasivam *et al*., 2012; DeCaprio, 2013; Sadasivam & DeCaprio, 2013). Upon entry into the cell cycle, p130 dissociates from the MuvB core and from E2F-dependent promoters, permitting activator E2Fs to transactivate G1-/S-phase genes (Longo & Finch, 2003; Sadasivam *et al*., 2012). The MuvB complex binds to B-MYB during S-phase and regulates expression of late S-phase genes. During G2, the MuvB–B-MYB complex recruits FOXM1, to these promoters (Lefebvre *et al*., 2010; Down *et al*., 2012; Sadasivam *et al*., 2012; Sadasivam & DeCaprio, 2013). While B-MYB undergoes phosphorylation-dependent, proteasome-mediated degradation, FOXM1 is activated through phosphorylation. Hyperphosphorylated FOXM1 remains bound to the MuvB complex and modulates expression of a range of genes such as Cyclin B, survivin, cdc25b phosphatase, and Aurora B kinase that are important for the G2–M transition (Lefebvre *et al*., 2010; Martinez *et al*., 2011; Down *et al*., 2012; Sadasivam *et al*., 2012). In senescence, both B-MYB and FOXM1 are lost and the MuvB complex recruits the transcriptionally repressive p130/p107–E2F4–DP complex, to form the DREAM complex, to restrict expression of both early and late cell cycle genes essential for cell cycle reentry.

During quiescence, the MuvB complex, comprised of LIN9, 37, 52, 54 as well as RBBP4, forms the DREAM complex upon association with p107/p130 RB-like pocket proteins and E2F4–DP1, which represses E2F-dependent genes (Fig. 1) (Sadasivam & DeCaprio, 2013). High p53 levels also induce a shift in MuvB core-associated proteins from B-MYB to p130/E2F4/DP1 (Quaas *et al*., 2012). However, upon cell cycle entry, p130 dissociates from the MuvB core and from E2F-dependent promoters, permitting activator E2Fs to transactivate G1-/S-phase genes (Longo & Finch, 2003; Sadasivam *et al*., 2012). The MuvB core then binds to B-MYB to form the B-MYB–MuvB complex that associates with the promoters of genes expressed later in S-phase. This complex in turn recruits FOXM1, a forkhead transcription factor, to these promoters during G2, enabling FOXM1 to activate expression of a range of genes such as Cyclin B, survivin, cdc25b phosphatase, and Aurora B kinase that are important for the G2–M transition (Fig. 1) (Lefebvre *et al*., 2010; Martinez *et al*., 2011; Down *et al*., 2012; Sadasivam *et al*., 2012; Sadasivam & DeCaprio, 2013). The MuvB core within these complexes is essential for targeting them to the promoters of specific sets of cell cycle-dependent genes (Sadasivam & DeCaprio, 2013).

FOXM1 is a highly conserved forkhead transcription factor vital for cell cycle progression (Katoh *et al*., 2013). Its expression is induced when quiescent cells reenter the cell cycle and reach a maximal level in S-phase that is maintained throughout G2 and mitosis. FOXM1 is downregulated upon replicative senescence in primary human fibroblasts (Hardy *et al*., 2005). Its expression is markedly reduced in cells from elderly patients as well as patients with progeria (Ly *et al*., 2000), and FOXM1-deficient mouse embryo fibroblasts undergo premature senescence (Wang *et al*., 2005). FOXM1 is a critical target of the CyclinD-CDK4/6 kinases; this phosphorylation leads to its stabilization and activation, thereby maintaining its expression and suppressing the levels of reactive oxygen species (Anders *et al*., 2011). More recent studies suggest that the activation of FOXM1 could have a role in reducing skin aging by inhibiting UVB-induced senescence (Ling *et al*., 2014). FOXM1 can also override genotoxic agent-induced senescence by modulating expression of DNA damage repair genes (Khongkow *et al*., 2013). As B-MYB is vital for the effects of FOXM1, this suggests that B-MYB may also play a role in preventing senescence through cell cycle progression. B-MYB is also implicated in the regulation of FOXM1 transcription, as depletion of B-MYB reduces FOXM1 expression and several other G2/M-specific genes in mouse fibroblasts (Down *et al*., 2012). Interestingly, B-MYB is also a target gene of FOXM1 and involved in complex cross-regulation with FOXM1 (Down *et al*., 2012). Although B-MYB exerts its main effects on the G2 stage of the cell cycle, it also increases transcription of genes important for S- and M-phase such as DNA topoisomerase by binding to MYB binding sites (MBSs) in their gene promoters (Martinez & DiMaio, 2011). B-MYB has been shown to be essential for S-phase progression and genomic stability in diploid and polyploid megakaryocytes, as B-MYB siRNAs reduced the number of cells that underwent DNA replication (García & Frampton, 2006). B-MYB also forms a complex with clathrin and filamin (MYB–Clafi complex) that is required for mitotic spindle function and therefore the G2–M transition (Yamauchi *et al*., 2008). Moreover, B-MYB is expressed in all dividing cells and acts to suppress the cyclin-dependent kinase inhibitor p16^INK4A^ (Huang *et al*., 2011). B-MYB therefore exerts extensive control over the cell cycle, whereas the effects of FOXM1 are limited to G2-/M-phase gene expression (Martinez & DiMaio, 2011). This suggests that B-MYB could be a more potent cell cycle progression gene than FOXM1, highlighting a potential role in preventing senescence.

### B-MYB and Cellular senescence

The role of B-MYB in cell cycle progression has led it to being commonly associated with tumor progression, leading to its role in cellular senescence being ignored. However, there is strong evidence to indicate that B-MYB is inhibited during senescence (Lin *et al*., 1994; Hardy *et al*., 2005; Martinez & DiMaio, 2011; Rovillain *et al*., 2011). Hardy and colleagues used cDNA microarrays to identify genes that were differentially expressed when conditionally immortal HMF3A fibroblasts underwent senescence growth arrest, followed by *in silico* promoter analysis of the differential genes and electrophoretic mobility shift assays (Hardy *et al*., 2005). Rovillain *et al*. (2011) extended this study using genome-wide expression profiling, in conjunction with inactivation of the p16 ^INK4A^-pRB and p53-p21^CIP1/WAF1/SDI1^ tumor suppressor pathways, in HMF3A cells, to identify genes that were downregulated upon senescence. This data, coupled to bioinformatic transcription factor analysis, revealed that B-MYB was one of the most highly downregulated transcription factors upon senescence. Upon further investigation, we found that ectopic expression of B-MYB bypasses senescence in HMF3A cells; in fact, B-MYB expression bypasses senescence more efficiently than FOXM1 (S.N. Mowla, P.S. Jat, unpublished data) suggesting that loss of B-MYB expression may have a causative role in senescence.

The first evidence implicating a causal role of B-MYB in cellular senescence was obtained by Lin *et al*. (1994) who constitutively expressed B-MYB in a human cell line, leading to a bypass of p53-p21^CIP1/WAF1/SDI1^_**-**_induced G1 arrest, even in the presence of CyclinE-CDK2 kinase inhibitors. Moreover, constitutive expression of B-MYB in BALB/c 3T3 fibroblasts reduced their growth factor requirement (Sala & Calabretta, 1992) and induced DNA synthesis in p107 growth-arrested human osteosarcoma cells (Sala *et al*., 1996). More definitive evidence, indicating a causal role of B-MYB in cellular senescence rather than quiescence, derives from Masselink *et al*. (2001) who showed that B-MYB expression could rescue oncogene-induced premature senescence caused by an activated *ras* oncogene in rodent cells. Moreover, siRNA silencing of B-MYB leading to reduced expression induced senescence in primary human foreskin fibroblasts and HeLa cervical cancer cells (Johung *et al*., 2007). Taken together, these results suggest that B-MYB impedes cellular senescence. However, it remains to be determined how B-MYB blocks senescence and how it is integrated into senescence-inducing pathways.

### Senescence pathways

**Figure 2:**
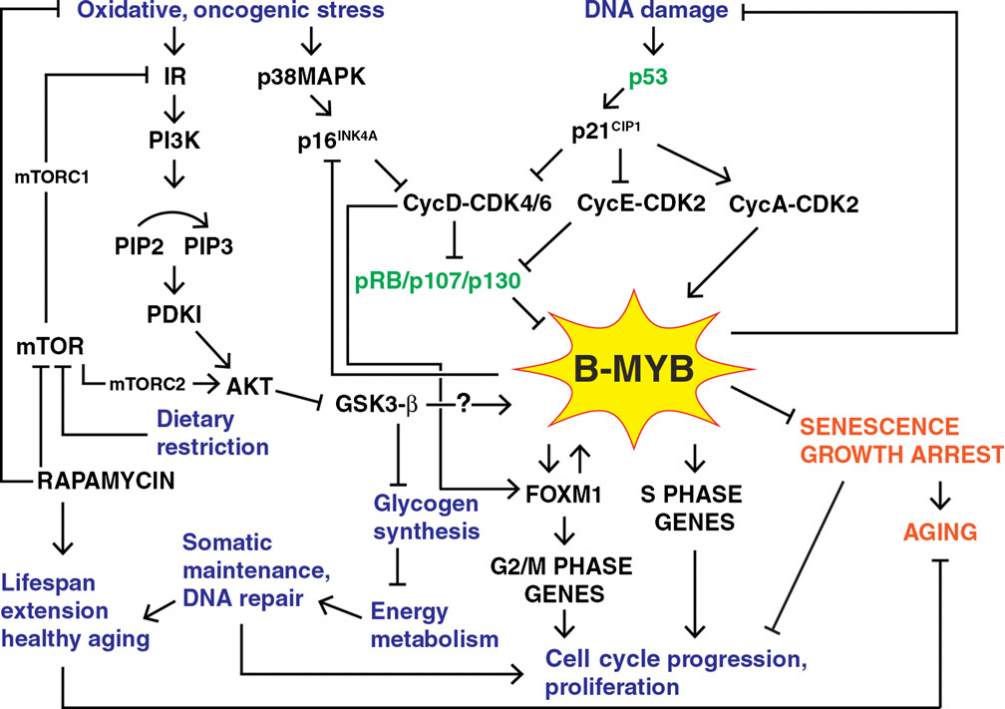
Schematic illustration of the pathways linking B-MYB to Cellular Senescence and Aging. This indicates how loss of B-MYB expression results in senescence growth arrest and thereby promotes aging. It also illustrates how oxidative/oncogenic stress, DNA damage, and dietary restriction may impinge upon senescence growth arrest and aging via B-MYB.

One way in which B-MYB represses senescence is through inhibition of the p16^INK4A^-pRB pathway (Fig. 2) (Sala & Calabretta, 1992; Yamauchi *et al*., 2008; Martinez *et al*., 2011). B-MYB expression is accompanied by Cyclin D1 expression (Sala & Calabretta, 1992). Other research indicates that B-MYB is also a transcriptional repressor, as its expression in embryonic lung fibroblasts leads to repression of p16^INK4A^, delaying cellular aging and increasing replicative lifespan; whereas knockdown of B-MYB expression increases expression of p16^INK4A^, thereby inducing cellular senescence (Huang *et al*., 2011). It can thus be suggested that B-MYB inhibits p16^INK4A^ leading to the activation of CyclinD-CDK4/6 and phosphorylation and inactivation of pRB. PRB has been reported to repress B-MYB expression during quiescence as well as senescence; however, the repression is much stronger during senescence due to the activation of the miR-29 and miR-30 families of microRNAs that strongly repress B-MYB expression (Martinez *et al*., 2011). Therefore, it can be hypothesized that extremely low levels of B-MYB lead to cellular senescence and this is achieved by negative feedback between pRB and B-MYB. When pRB represses B-MYB to moderately low levels, this leads to quiescence, but when pRB also activates microRNAs resulting in very low levels of B-MYB and therefore less inhibition of pRB, this leads to senescence.

Our data indicates that the effects of B-MYB expression on bypassing senescence are greater than inhibition of pRB by adenovirus E1A, HPV E7, and E2F-DB, suggesting that B-MYB must also be acting upon other targets in senescence. One possible candidate is p53 because, as mentioned above, one critical activity p53 engages in, to arrest the cell cycle, is shifting the MuvB core-associated proteins from B-MYB to p130/E2F4/DP1 (Quaas *et al*., 2012). Our data reveal that ectopic expression of B-MYB bypasses senescence in HMF3A cells in which activation of p53 is the major pathway for inducing senescence (S.N. Mowla, P.S. Jat, unpublished data). However, questions still remain as to how B-MYB inhibits activated p53 and whether it inhibits the transcription of p53 or acts downstream. One possibility is that high levels of B-MYB may prevent the displacement of B-MYB by p130 in MuvB complexes.

Taken together, it can be postulated that high levels of B-MYB are required for cell proliferation, aided via its effects on FOXM1, and that the levels of B-MYB may therefore be a deciding factor in switching the fine balance between proliferation, quiescence, and cellular senescence. Shedding further light on the MuvB complex, its interaction with B-MYB and FOXM1 and whether these three components are present in the same complex simultaneously would be important in determining how B-MYB interacts and engages with senescence pathways. However, B-MYB may also be interacting with other as yet unknown pathways involved in senescence.

### Cell death

B-MYB has also been reported to play a role in cell death. Most studies suggest that B-MYB promotes cell survival through the regulation of apoptotic genes (Sala, 2005). For example, overexpression of B-MYB in CTLL-2 cells causes resistance against apoptosis induced by DNA-damaging agents such as doxorubicin (Grassilli *et al*., 1999). This is in part due to an increased transcription of pro-survival genes, such as BCL2 and inhibition of apoptotic genes (Grassilli *et al*., 1999; Sala, 2005), but is also due to an increase in resistance to DNA damage in B-MYB expressing cells. B-MYB has been shown to increase genomic stability, as cells lacking B-MYB are more sensitive to DNA damage induced by UV radiation (Ahlbory *et al*., 2005). Equally, neuroblastoma cells expressing high levels of B-MYB are resistant to UV-induced apoptosis (Schwab *et al*., 2007). As DNA damage is a key activator of cellular senescence, the role of B-MYB in protecting against such damage is therefore consistent with a role of B-MYB in impeding senescence.

### Development

Although senescence was originally identified as a stable cell cycle arrest, it has now been shown that senescence is also a programmed developmental mechanism that contributes toward mammalian embryonic development (Muñoz-Espín *et al*., 2013; Storer *et al*., 2013). B-MYB is robustly expressed in embryonic stem cells and has been reported to sustain the self-renewal capacity of such cells (Zhan *et al*., 2012) by promoting the genomic stability and survival of these cells. Silencing of B-MYB in murine embryonic stem cells results in delayed transit through G2/M, severe mitotic spindle and centrosome defects, polyploidy, and differentiation-associated cell death (Tarasov *et al*., 2008). B-MYB is therefore critical to the establishment of inner cell mass through cell cycle progression and is vital in sustaining stem cell populations and self-renewal capacity, two vital factors in the maintenance of tissue function, and thus healthy aging.

### Nutrient and metabolic signaling

B-MYB has very recently been implicated in nutrient signaling, and this may be an important pathway that connects it to anti-aging signaling (Fig. 2). During energy metabolism following food consumption, insulin activates the receptor tyrosine kinase, insulin receptor (IR), which then binds to the PTB domain of insulin receptor substrate 1 (IRS1), causing the recruitment of PI3K to the cell surface. Here, PI3K (I) catalyses the addition of a phosphate on the 3′ position of PI(4,5)P2 using ATP to generate PI(3,4,5)P3, which in turn recruits the serine/threonine kinase AKT (PKB) to the cell membrane (Korkolopoulou *et al*., 2012). PI (3,4,5)P3 brings AKT and the activating kinase of AKT, PDK1, in close proximity, enabling PDK1 to partially activate AKT; mTORC2, then works in conjunction with PDK1 to fully activate AKT. This results in the phosphorylation and inactivation of glycogen synthase kinase 3β (GSK3β), which normally inhibits glycogen synthesis; activation of this pathway hence results in the storage of energy in the form of glycogen (Morgensztern & McLeod, 2005). Interestingly, the bulk of literature concerning anti-aging currently revolves around the inhibition of this pathway through dietary restriction or rapamycin (Fig. 2); these are currently among the hottest topics in aging research.

Rapamycin, an inhibitor of mTOR and therefore the mTOR pathway, can extend lifespan *in vitro* and *in vivo*. For example, feeding mice with rapamycin dramatically extends both female and male lifespan (Harrison *et al*., 2009; Livi *et al*., 2013). Such treatment has also been reported to reduce age-related disease as treating 24-month-old mice with rapamycin-reversed age-related heart dysfunction (Flynn *et al*., 2013). However, highly significant increases in longevity induced by the inhibition of the mTOR pathway are not limited to mice, rather, such effects have also been reported in other organisms (Jia *et al*., 2004; Kapahi *et al*., 2004). More interestingly, rapamycin reduces oxidative stress and premature senescence *in vitro*, leading to an increase in replicative lifespan, thereby connecting this pathway to cellular senescence and as a result, potentially to B-MYB (Li *et al*., 2012).

A potential pathway as to how mTOR could impinge upon the B-MYB pathway has recently been discovered in Arabidopsis, although it remains to be identified in mammals. It has been found that B-MYB is a substrate for the GSK3-like kinase, BIN2, and phosphorylation by BIN2 stabilizes B-MYB (Ye *et al*., 2012). As mTOR can inhibit GSK3β, either through mTORC2 acting in conjunction with AKT or by mTORC1 acting further upstream, inhibition of mTOR using rapamycin could activate B-MYB, leading to an extension in healthspan and lifespan. Other studies suggest that mTOR may activate pRB by regulating upstream CDK activity through p27^KIP1^ and Cyclin D1 (Mita *et al*., 2003) and as described earlier, pRB and B-MYB are negative regulators of one another. The inhibition of B-MYB downstream of mTOR, via pRB activation, further supports the hypothesis that mTOR inhibition activates B-MYB. Although the GSK3β pathway remains to be elucidated in animals, the Arabidopsis results suggest a potential pathway linking mTOR to B-MYB.

One currently popular theory, as to how rapamycin extends lifespan, is somatic maintenance, which suggests that the inhibition of mTOR leads to a reallocation of energy into the maintenance of somatic cells (Fig. 2) (Masoro & Austad, 1996). As described above, B-MYB acts to maintain genome stability and provide resistance against DNA damage, thereby inhibiting cellular senescence. The energy required for the maintenance of somatic cells by B-MYB could potentially be gained through inhibition of mTOR, thereby coupling energy metabolism and senescence signaling, two major pathways on the road to aging.

## Conclusions

B-MYB mediates a multitude of signaling pathways that affect cellular senescence, cell growth, metabolism, and cell death and lies at the hub of numerous signaling networks involved in these cellular behaviors. Moreover, the levels of B-MYB regulated through p53 and pRB determines the fine balance between proliferation, quiescence, and senescence, making B-MYB a determinant of cell fate. This poses the question that the increase in lifespan seen upon inhibition of mTOR may be due to the upregulation of B-MYB and that the reallocation of energy achieved from the inhibition of this pathway may be what permits B-MYB to have an antisenescence role, in combination with its pro-cell cycle activities. However, due to the oncogenic potential of B-MYB, very little research has been performed to investigate the role of B-MYB in aging. Future studies *in vitro* and *in vivo,* on how B-MYB integrates into signaling pathways, in particular with mTOR in animal models, its upstream and downstream effectors, and the effects of B-MYB on cellular senescence and longevity, will be a promising new avenue for anti-aging research.
